# Intersection of inflammation and viral replication: the central role of MAPK signaling in viral respiratory infections

**DOI:** 10.3389/fmicb.2025.1735254

**Published:** 2026-01-22

**Authors:** Ralph A. Tripp, Les P. Jones, David E. Martin

**Affiliations:** 1 Department of Infectious Diseases, University of Georgia, Athens, GA, United States; 2 TrippBio, Inc., Jacksonville, FL, United States

**Keywords:** antiviral therapy, ERK, inflammation, JNK, MAPK, p38, signaling cascades, viral replication

## Abstract

The mitogen-activated protein kinase (MAPK) pathway is a vital cellular signaling cascade that viruses exploit. When activated by viruses, this pathway also initiates the host’s inflammatory response. This pathway has a crucial role in viral respiratory infections, serving as a key intersection where viral replication and host inflammation are coordinated. Some viruses activate this pathway to enhance their own replication while also triggering inflammatory responses in the host. Understanding this intersection is essential because therapeutic agents could target the same pathway to inhibit both viral replication and inflammation. This perspective considers targeting the MAPK pathway as a potential way to treat viral respiratory infections by suppressing viral replication and reducing inflammation.

## Introduction

Inflammation has a crucial role in regulating viral respiratory infections. It triggers an immune response that helps eliminate the virus. Severe inflammation can harm lung tissue, and some viruses have evolved mechanisms to inhibit anti-inflammatory signals, enabling prolonged replication. Notably, inflammation activates immune cells and directs them to the site of viral infection to eliminate infected cells. However, this process can also be harmful, as off-target effects can damage non-infected lung tissues, contributing to complications such as pneumonia. Intriguingly, certain viruses suppress the anti-inflammatory response, allowing inflammation to escalate. Understanding these complex interactions is essential for developing therapies that manage the harmful effects of inflammation while maintaining the immune system’s ability to eliminate the virus.

Mitogen-activated protein kinase (MAPK) pathways are vital cellular signaling cascades that, when activated, initiate a three-tiered kinase cascade leading to a wide range of cellular responses. They are activated by viruses and inflammatory signals and play roles in processes such as cell proliferation, apoptosis, and the immune response. The MAPK family includes p38 kinase, c-Jun N-terminal kinase (JNK), and extracellular-regulated kinase (ERK) pathways. Viral and inflammatory signaling pathways come together with MAPKs to regulate gene expression and produce cytokines (Chaudhary et al., 2023). Additionally, viruses can hijack MAPK pathways to aid in their replication and pathogenesis (Higgins et al., 2023). Viral signaling can activate MAPK pathways through pattern recognition receptors (PRRs) that detect infection, triggering an immune and inflammatory response (Thompson et al., 2011). The induction of pro-inflammatory cytokines begins with PRR signaling, in which pathogen-associated molecular patterns (PAMPs) recognize a target molecule, such as a virus. Upon detection, these receptors trigger intracellular signaling cascades, including those involving the NF-κB pathway, which lead to the production of pro-inflammatory cytokines such as TNFα, IL-1, and IL-6. These cytokines affect the immune response by enhancing vascular permeability, recruiting immune cells, and activating them to control and eliminate the pathogen. Viruses exploit MAPK pathways to promote their replication and survival (Kumar et al., 2018). Viral proteins can affect MAPK signaling and lead to pathogenesis. For example, Epstein-Barr virus (EBV) latency proteins are known to interact with the MAPK-ERK pathway, promoting infection, cell migration, and viral latency (Šimičić et al., 2024). Additionally, viruses can alter MAPK signaling to regulate inflammatory responses and can lessen the pro-inflammatory response, particularly via the p38 pathway. Notably, MAPK signaling regulates inflammasomes, essential components of the innate immune system (Shin et al., 2021).

This Perspective hypothesizes that inflammation and viral replication contribute to disease severity and offers new therapeutic opportunities. Inflammation and viral replication are interconnected through MAPK signaling. MAPK pathways are involved in the inflammatory response and can also be manipulated by viruses to enhance viral replication. This complex interplay suggests that disrupting the MAPK pathway could reduce both viral replication and inflammation, leading to less severe disease. Those drugs specifically targeting the MAPK pathway could offer a novel approach to treating viral infections by simultaneously reducing viral load and mitigating harmful inflammation.

## Overview of MAPK signaling pathways

**FIGURE 1:**
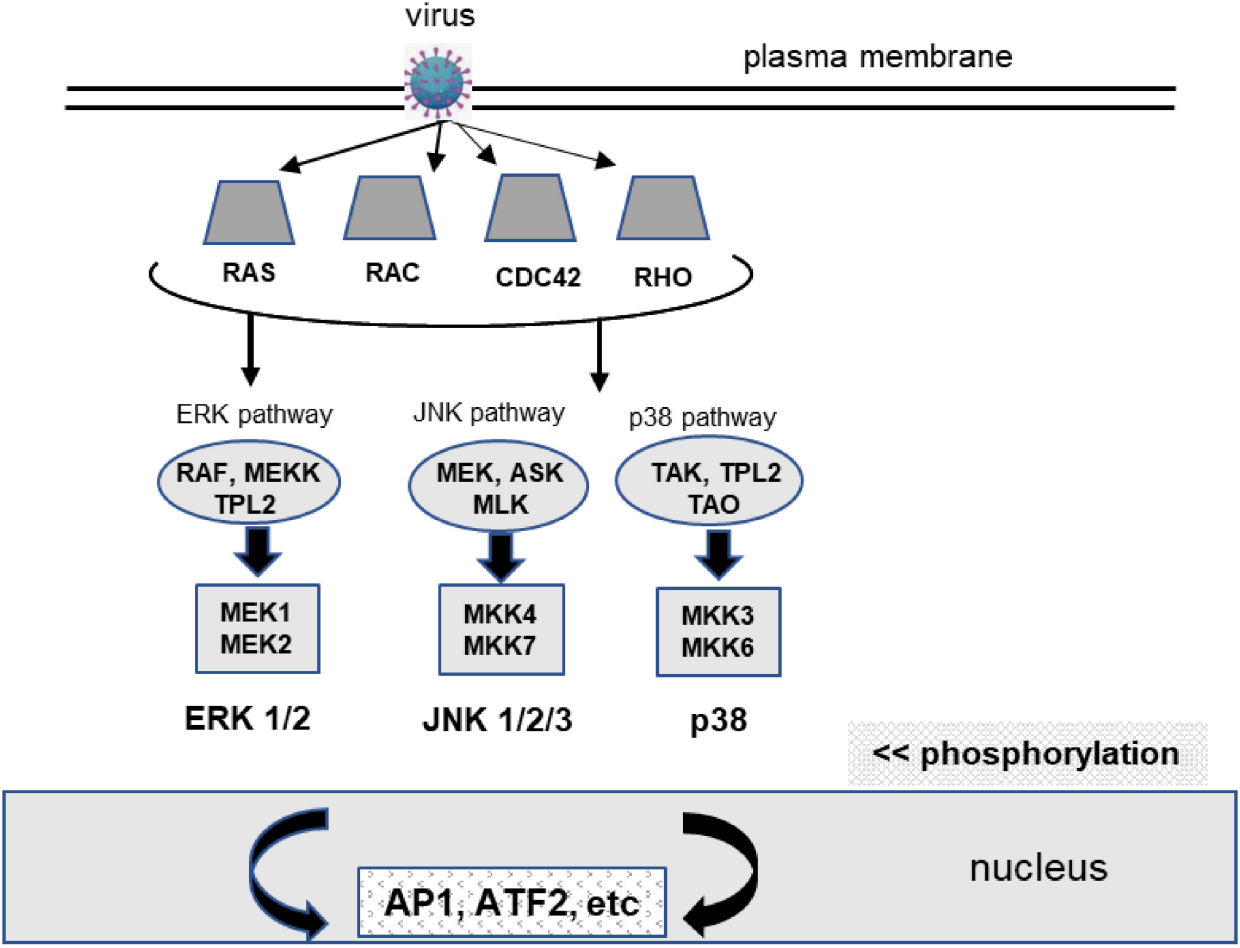
Extracellular-regulated kinase (ERK), JNK, and p38 pathways in MAPK signaling. Upon external stimulation, e.g., a virus, receptor tyrosine kinases, a family of transmembrane proteins that function as cell-surface receptors, activate the three-tiered kinase module. Activated MAPKs translocate to the nucleus and trigger cellular responses. RAF kinase activates MEK, which then phosphorylates and activates ERK. MEKK phosphorylates and activates MEK and the kinase TMPL2. ASK is a MAP3K that activates the JNK and p38 pathways. TPL2 regulates ERK and p38, which drive the production of pro-inflammatory mediators and regulate immune cell function. Proinflammatory cytokines activate TAK1 and mediate activation of NF-κB, JNK, and p38. ERK primarily regulates proliferation and survival, whereas JNK and p38 respond to stress and inflammatory stimuli. Significant crosstalk occurs, particularly between JNK and p38, coordinating responses to stress and infection (Yan et al., 2024; Bachstetter and Van Eldik, 2010).

The purpose of the MAPK pathways is to transmit signals from the cell surface to the nucleus, regulating crucial cellular activities such as growth, differentiation, and apoptosis. These pathways consist of three kinases: MAPKKK (Mitogen-Activated Protein Kinase Kinase Kinase), MAPKK (Mitogen-Activated Protein Kinase Kinase), and MAPK (Mitogen-Activated Protein Kinase) (Kostenko et al., 2011). ERK, JNK, and p38 are activated by various stimuli, such as growth factors or stress, and are responsible for specific cellular functions. For instance, ERK is primarily involved in cell proliferation, while JNK and p38 are associated with inflammation and apoptosis (Yue and López, 2020). The signaling process begins when a factor binds to a receptor tyrosine kinase on the cell surface. This binding engages adaptor proteins that activate Ras proteins, a family of GTPases that function as “on/off” switches in cell signaling pathways. Once activated, Ras triggers a three-tiered cascade of kinases. Specifically, MAPKKK, a crucial link in the MAPK signaling pathway, relays signals from MAPKKK to MAPKK, and then to MAPK (Figure 1).

A fundamental feature of MAPK signaling pathways involves the family of dual-specificity phosphatases (DUSPs), which are potent regulators of biological processes and act as negative regulators by dephosphorylating and inactivating MAPKs (Huang and Tan, 2012). DUSPs deactivate MAPKs through dephosphorylation, forming a feedback loop that limits signaling. Twenty-five DUSPs exist, classified as typical or atypical depending on MAPK docking domains. Their expression is induced by MAPK activation, and activity varies across cell types and stimuli (Seternes et al., 2019; Huang and Tan, 2012). For example, DUSP1 can target ERK, p38, and JNK, depending on the context, fine-tuning MAPK activity to maintain appropriate responses to infection and stress. DUSPs are produced through gene expression, triggered by signals such as growth factors or cellular stress, leading to the activation of MAPKs. This activation, in turn, causes the DUSP genes to be transcribed and translated into DUSP proteins, creating a feedback loop that dephosphorylates and inactivates the MAPKs (Lang and Raffi, 2019). There are inducible nuclear MAPK phosphatases, e.g., DUSP1, DUSP2, DUSP4, and DUSP5, the cytoplasmic, ERK-specific MKPs DUSP6, DUSP7, and DUSP9, and DUSP8, DUSP10, and DUSP16 that are found in both the cytoplasm and nucleus (Seternes et al., 2019). Importantly, the expression levels and regulation of DUSPs are cell type and context-specific and differ depending on whether they are constitutive or inducible (Pérez-Sen et al., 2019). This complex regulatory network highlights the importance of DUSPs, which serve as fine-tuners of MAPK signaling and are critical to these pathways.

## MAPK activation in viral respiratory infections

Viruses enter host cells primarily by penetration (for non-enveloped viruses) or by fusion (for enveloped viruses) (Klasse et al., 1998; Cossart and Helenius, 2014). During entry, the stimulation of Toll-like receptors (TLRs) activates MAPK signaling pathways, which regulate TLR transcription and activate various transcription factors. Once inside the host cell, viruses are transported through the cytoplasm as nucleoprotein complexes. Early activation of MAPK occurs shortly after viral infection, often triggering immediate-early viral gene expression and creating a cellular environment conducive to replication, such as promoting cell cycle progression. Late MAPK activation occurs later in the infection cycle and is essential for the synthesis of viral structural proteins and the assembly and release of progeny viruses (Motamedi et al., 2020). This temporal distinction is crucial for the virus to exploit the host cell machinery and its replication.

Each respiratory virus activates MAPKs in distinct ways. For example, influenza A virus (IAV) relies on ERK signaling, partly via hemagglutinin accumulation at the plasma membrane (Dey and Mondal, 2024). Respiratory syncytial virus (RSV) relies on p38 MAPK via TLR4 (Marjuki et al., 2011; McCaskill et al., 2017), while suppressing innate immunity by sequestering NF-κB p65 in cytoplasmic bodies (Jobe et al., 2020). Viruses may also induce MAPK activation indirectly through factors such as stress, inflammasomes, or TLRs (Bauernfeind and Hornung, 2013; Thompson et al., 2011; Shi et al., 2023). MAPK signaling during RSV infection influences chemokine production (e.g., RANTES) and recruits immune cells. MAPK pathways may also be activated indirectly via inflammasomes or broader cellular stress responses, contributing to viral replication efficiency.

## MAPK-dependent inflammatory responses

Mitogen-activated protein kinases regulate inflammation through multiple mechanisms, including activating AP-1 transcription factors that induce cytokine genes (Chen et al., 2018). p38 enhances cytokine production by stabilizing inflammatory mRNAs (Winzen et al., 1999), and MAPKs and NF-κB cooperate during PRR signaling, shaping the intensity and duration of inflammation (Gottschalk et al., 2016). MAPK pathways are essential for recruiting immune cells by activating inflammatory gene expression and promoting the release of chemokines and cytokines that attract immune cells to sites of infection.

## MAPK signaling and viral replication

There is ample evidence that MAPKs play a significant role in various viral processes, including transcription, translation, assembly, and viral release. The JNK and p38 pathways activate transcription factors, such as the AP-1 family, by phosphorylation, thereby altering gene expression essential for viral replication (Peng et al., 2014; Wei et al., 2009; McLean and Bachenheimer, 1999). Inhibiting these pathways significantly reduces viral mRNA levels for specific viruses, as these MAPKs are crucial for viral protein synthesis. For example, JNK has been shown to phosphorylate the nucleocapsid protein of HCoV-229E, which is necessary for replication (Bruggemann et al., 2025). Additionally, inhibiting both p38 and JNK decreases viral protein expression (Wei et al., 2009). Some viruses that utilize these pathways include human adenoviruses (Nestic et al., 2021), which require activation of the ERK, p38, and JNK pathways for replication. Also, for HIV-1, the JNK and p38 MAPK pathways are essential for both virus infection and replication (Muthumani et al., 2004).

## Inflammation and viral replication are a positive feedback cycle

The inflammation–viral replication loop is a positive feedback cycle in which a virus triggers inflammation, which, in turn, promotes further viral replication, exacerbating the inflammatory response. This cycle can result in excessive inflammation, including a cytokine storm, which can lead to tissue damage and worsen the severity of viral infections. However, some immune mechanisms within this loop can aid in viral clearance by amplifying the inflammatory response, although dysregulation can lead to harmful outcomes. The cycle begins with an initial viral infection that activates an immune response. The immune system releases inflammatory mediators such as TNFα, IL-1β, and interferons (IFNs) to combat the disease. Unfortunately, the virus can manipulate this inflammatory response to its advantage. Inflammatory mediators attract additional immune cells, which in turn release more chemokines and cytokines, creating a feed-forward loop that intensifies the inflammatory response. This exaggerated inflammation creates an environment that favors increased viral replication, leading to an ongoing cycle of heightened viral replication and further inflammation, sometimes referred to as a cytokine storm.

Mitogen-activated protein kinases phosphorylate key transcription factors (Balendran et al., 2023), which then translocate to the nucleus, inducing the expression of genes encoding inflammatory mediators. MAPK activation supported both inflammation and increased viral replication by triggering the expression of inflammatory genes and providing the cellular machinery needed for viral replication.

## Therapeutic implications

Targeting MAPK pathways offers a host-directed antiviral strategy by modulating both viral replication and pathological inflammation. MAPK pathway inhibitors with antiviral activity against respiratory viruses are supported by a small but growing body of pre−clinical and early clinical evidence, most robustly for the MEK inhibitor zapnometinib (ATR−002) in influenza and COVID−19 and for clinically pre−evaluated p38α/β inhibitors in SARS−CoV−2 models (Schreiber et al., 2022; Koch-Heier et al., 2022, 2024; Faist et al., 2023; Rohde et al., 2023). These data collectively demonstrate that targeting host MAPK signaling can constrain viral replication while modulating pathogenic inflammation, although definitive, late−phase efficacy data in humans remain limited (Faist et al., 2022; Hein and Weissman, 2022; Rohde et al., 2023).

### MEK and p38 inhibition as a host−directed antiviral strategy

Pre−clinical studies with zapnometinib, a selective MEK1/2 inhibitor that blocks Raf–MEK–ERK signaling, have shown potent inhibition of influenza A and B virus replication in cell culture and animal models, with reduced viral titers and attenuation of lung inflammation (Koch-Heier et al., 2024; Hein and Weissman, 2022; Schreiber et al., 2022). Mechanistically, MEK inhibition prevents nuclear export of influenza ribonucleoprotein complexes and dampens virus−induced pro−inflammatory cytokine production, thereby targeting both viral replication and the MAPK−driven inflammatory response that contributes to lung injury (Koch-Heier et al., 2024; Schreiber et al., 2022).

These pre−clinical findings have led to clinical development of zapnometinib as a host−targeted antiviral for influenza and COVID−19, with oral dosing designed to achieve MEK inhibition sufficient to reduce ERK phosphorylation in circulating and tissue immune cells (Koch-Heier et al., 2022; Schreiber et al., 2022). In the phase 2 RESPIRE trial in hospitalized adults with moderate to severe COVID−19 (NCT04776044), zapnometinib plus standard of care was well tolerated and achieved substantial MEK pathway inhibition. Exploratory efficacy analyses showed higher odds of improved clinical status and a trend toward earlier discharge–particularly in patients with greater baseline severity - supporting further evaluation in larger randomized studies (Rohde et al., 2023).

SARS−CoV−2 strongly activates p38 MAPK signaling and exploits p38β to support efficient replication in human cells, highlighting p38 as an attractive host target (Higgins et al., 2023; Faist et al., 2022). In a comprehensive pre−clinical evaluation, the selective and clinically pre−evaluated p38α/β inhibitors PH−797804 and VX−702 markedly reduced expression of pro−inflammatory cytokines such as IL−6, CXCL8, CXCL10, and TNF in infected primary human lung explants and epithelial organoids, while preserving the type I interferon−dependent antiviral response of the lung epithelial barrier (Faist et al., 2023).

p38 inhibition with PH−797804 or VX−702 also showed synergy with the nucleoside analogs remdesivir and molnupiravir, producing an additional 2–3 log reduction in SARS−CoV−2 titers and high drug−synergy scores *in vitro*, which suggests that MAPK blockade can potentiate direct−acting antivirals without compromising innate antiviral signaling (Faist et al., 2023). Although PH−797804 and VX−702 have been evaluated clinically in non−viral inflammatory indications, dedicated clinical trials of p38 inhibition for COVID−19 or other respiratory virus infections have not yet been reported, so their antiviral potential currently rests on pre−clinical proof−of−concept data (Faist et al., 2023).

### Additional MAPK−targeted approaches in respiratory virus infection

Beyond direct MEK and p38 inhibitors, upstream modulation of MAPK activation provides further evidence that these pathways are functionally required for respiratory virus replication (Faist et al., 2023; Broadbent et al., 2021). In well−differentiated primary airway epithelial cultures infected with RSV, pharmacologic inhibition of p38 MAPK using SB203580 significantly reduced RSV titers over 96 h compared with vehicle controls. However, the effect size was smaller than that observed with TLR4 inhibition by TAK−242, consistent with p38 acting as a key downstream effector within the TLR4−proximal signaling cascade (Broadbent et al., 2021). In the same model, p38 inhibition decreased RSV−induced interferonλ1 secretion, underscoring a complex balance between antiviral and pro−inflammatory signaling downstream of MAPK activation in the airway mucosa (Broadbent et al., 2021).

Collectively, these findings indicate that MAPK inhibitors can exert broad antiviral effects across multiple respiratory viruses by targeting shared host signaling nodes rather than virus−encoded proteins (Schreiber et al., 2022; Koch-Heier et al., 2022, 2024; Faist et al., 2023). At the same time, the dual roles of ERK and p38 in cell survival, innate antiviral defense, and cytokine production emphasize the need for careful dose selection, treatment timing, and rational combination strategies to optimize the therapeutic window and minimize on−target toxicities when repurposing MAPK inhibitors developed for oncology or chronic inflammatory indications for acute viral infections (Bahar et al., 2023; Faist et al., 2022; Koch-Heier et al., 2022).

## Discussion

An unresolved question about the MAPK pathway is how it achieves signaling specificity. Cytoplasmic serine/threonine kinases transduce extracellular signals into regulatory events that influence cellular responses (Craig et al., 2008; Manna and Stocco, 2011). The activation of one kinase triggers the activation of several downstream kinases, which then regulate transcription factors and affect gene function. This arrangement allows the kinase cascade to be amplified and integrated according to the cellular context. MAPK cascades amplify extracellular signals, yet how MAP3Ks and MAP2Ks selectively activate downstream MAPKs remains poorly understood. Crosstalk with PI3K and other pathways complicates understanding the network. Different isoforms (p38α, β, γ, δ; JNK; ERK) may have distinct roles in immunity, stress responses, and development (Garcia-Hernandez et al., 2021).

The therapeutic window for MAPK inhibitors varies depending on the condition and target, as inhibiting the pathway can lead to side effects. The therapeutic benefit relies on a careful balance between pathway inhibition and normal physiological processes, possibly requiring combination therapies to overcome drug resistance. The therapeutic window is narrow because the MAPK pathway is involved in many essential cellular functions. This pathway is also crucial for normal development and function. MAPKs serve as a double-edged sword in viral infections: they facilitate antiviral defense while viruses exploit them to replicate. Their central roles in inflammation, viral replication, and stress responses make them promising targets for host-directed antiviral therapy.
